# Comprehensive evaluation of candidate reference genes for real-time quantitative PCR (RT-qPCR) data normalization in nutri-cereal finger millet [*Eleusine Coracana* (L.)]

**DOI:** 10.1371/journal.pone.0205668

**Published:** 2018-10-15

**Authors:** Palakolanu Sudhakar Reddy, Mahamaya G. Dhaware, Dumbala Srinivas Reddy, Bommineni Pradeep Reddy, Kummari Divya, Kiran K. Sharma, Pooja Bhatnagar-Mathur

**Affiliations:** International Crops Research Institute for the Semi-Arid Tropics (ICRISAT), Patancheru, Hyderabad, Telangana, India; CSMCRI, INDIA

## Abstract

Finger millet (*Eleusine coracana* L.) is an annual herbaceous self-pollinating C_4_ cereal crop of the arid and semi-arid regions of the world. Finger millet is a food security crop proven to have resilience to changing climate and scores very high in nutrition. In the current study, we have assessed sixteen candidate reference genes for their appropriateness for the normalization studies in finger millet subjected to experimental regimes and treatments. Ten candidate reference genes (*GAPDH*, *β-TUB*, *CYP*, *EIF4α*, *TIP41*, *UBC*, *G6PD*, *S24*, *MACP* and *MDH*) were cloned and six (*ACT*, *ELF1α*, *PP2A*, *PT*, *S21* and *TFIID*) were mined from the NCBI database as well as from the literature. Expression stability ranking of the finger millet reference genes was validated using four different statistical tools i.e., geNorm, NormFinder, BestKeeper, ΔCt and RefFinder. From the study, we endorse *MACP*, *CYP*, *EIF4α* to be most stable candidate reference genes in all ‘tissues’, whereas *PT*, *TFIID*, *MACP* ranked high across genotypes, *β-TUB*, *CYP*, *ELF1α* were found to be best under abiotic stresses and ‘all samples set’. The study recommends using minimum of two reference genes for RT-qPCR data normalizations in finger millet. All in all, *CYP*, *β-TUB*, and *EF1α*, in combination were found to be best for robust normalizations under most experimental conditions. The best and the least stable genes were validated for confirmation by assessing their appropriateness for normalization studies using *EcNAC1* gene. The report provides the first comprehensive list of suitable stable candidate reference genes for nutritional rich cereal finger millet that will be advantageous to gene expression studies in this crop.

## Introduction

Finger millet, *Eleusine coracana* (L.) Gaertn, is a nutri-cereal grown for food and feed in Africa and South Asia regions of the world. This tiny grain displays high variability in the mineral composition and has superior nutritional qualities compared to other cereal crops including rice and wheat used as a health food, and in bakery [1–3]. Besides being a stable source of nutrition to millions of people in semi-arids, finger millet is quite a resilient crop, and hence has attracted lot of attention of researchers for studying its genetics, genomics for its improvement [4–8].

Finger millet is vulnerable to both abiotic and biotic stresses, with blast disease being a primary constraint [9,10] and drought and salinity stresses affecting the crop production systems and economics [11,12]. To overcome these stresses, there is a need to deploy beneficiary regulatory and structural genes through functional genomics approaches. Towards this RT-qPCR technology offers promise for studying the function of desired genes with high sensitivity, precision, simplicity and robustness [13–15]. Nonetheless, there are certain limitations to this technology essentially due to lack of the appropriate reference gene (s), which further effects the threshold (Cq) values and eventually affect the precision of the expression [16,17]. Experiment-to-experiment difference depends on the reference genes expression, which is quashed through the process of ‘normalization’. Across most species, the most commonly used reference genes (RG) have been housekeeping genes (HKG) with the fundamental supposition that their expression levels remain unchanged regardless of the condition or nature of the sample during the course of the experiments [18,19]. The trustworthiness of the RT-qPCR data trusts on stable expression of the candidate reference genes across the conditions irrespective of the samples [20–22].

So far most of the finger millet gene-expression studies have relied on conventional reference genes including *EcActin* [23–28], *EcEF-1a* [24], and *EcTUB* [29–34] for normalization studies under various experimental conditions. However, several reports have confirmed the instability in expression of conventional reference genes under various experimental conditions [35,36]. It has now been established that most of the reference genes within the plant demonstrate variable expressions from experiment to experiment and sample to sample [20,21,36]. Therefore, it is prudent to experimentally validate the appropriateness of reference genes in the target species rather than its universally acceptance across species [37, 38]. A number of evaluation approaches have been adopted in plant species to verify these inconsistencies in expression of conventional reference genes through systematic studies in *Arabidopsis* [35], potato [39], barley [40], sorghum [21], *Setaria viridis* [41]; melon [42], pearl millet [20], goose grass [43], foxtail millet [44], soybean [45] and ryegrass [46].

Considering that until now there is no work done in this direction for finger millet, the present study was undertaken for assessment of sixteen reference genes, including, *Actin* (*ACT*), *Beta Tubulin* (*β-TUB*), *Cyclophilin* (*CYP*), *Eukaryotic Initiation factor 4A* (*EIF4α*), *Elongation factor 1-alpha* (*EF1α*), *Glyceraldehyde-3-phosphate dehydrogenase* (*GAPDH*), *Glucose-6-phosphate 1-dehydrogenase* (*G6PD*), *MalonylCoA-Acyl Carrier protein* (*MACP*), *Malate dehydrogenase* (*MDH*), *40S ribosomal protein* (*S24*), *Serine/threonine-Protein Phosphatase* (*PP2A*), *Phosphate transporter protein* (*PT*), *Ribosomal protein* (*S21*), *Transcription initiation factor* (*TFIID*), *Tonoplast intrinsic proteins-like protein* (*TIP41*) and *Ubiquitin Protein Isoform C* (*UBC*) as reference genes for RT-qPCR in finger millet. The expression stability of the sixteen reference genes across the regime of diverse experiments was evaluated using geNorm [19], NormFinder [47], BestKeeper [48] and ΔCt [49] statistical tools. To our knowledge it is the first attempt on a systematic evaluation of the reference genes in *Eleusine coracana* (L.). The conclusion of the study definitely will advantage those experiments which involve gene expression studies in finger millet species and also in other closely related millets.

## Materials and methods

### Plant material and abiotic stress treatments

Finger millet (variety GPU 28) has been used for the different abiotic stress treatments and tissue/organ collection. GPU 28 seeds were sown in pots comprising red soil mixture (3:2:1 clay:sand:manure) and grown in a greenhouse with day/night average temperatures of 27/22°C and relative humidity of 70–80%. Five major abiotic stresses (salt, cold, heat, drought and ABA) and different tissues (seedling, leaf, root, panicle, and mature seed) were harvested [20,21]. Finger millet cultivars contrasting for drought stress response (Tolerant—IE 4073, IE 4797 and GPU 28; sensitive—IE 5106 and IE 2572) [50] were grown under greenhouse conditions and imposed progressive drought stress after 28 days and leaf tissues were collected when normalized transpiration ratio (NTR) reached at 0.1. All the samples were collected in triplicates and straightaway snap-frozen in liquid nitrogen and stored at -80°C till RNA isolation.

### Sequence mining, cloning and RT-qPCR primers designing

Sixteen candidate reference genes, including *ACT*, *ELF1α*, *PP2A*, *PT*, *S21* and *TFIID* were retrieved from the available sequence information of finger millet deposited in the NCBI database. Remaining ten-candidate reference genes were cloned from the sequence information of different plant species, including pearl millet (*β-TUB*, *S24*, *CYP* and *GAPDH*), chickpea (*EIF4α*, *TIP41* and *UBC*), groundnut (*G6PD*) and sorghum (*MACP* and *MDH*). Two micrograms of finger millet total RNA was used for cDNA synthesis (Invitrogen, USA) and PCR amplification was carried out with respective gene specific primers according to the manufacturer’s instructions (Invitrogen). The amplified PCR products were cloned into the pCR4.0-TOPO vector (Invitrogen) and sequenced. RT-qPCR primers were designed using primer 3.0 software (http://bioinfo.ut.ee/primer3-0.4.0/) [51] with default settings with the following considerations: (a) product size: 90–170 bp; (b) primer length: 18–24 bp and (c) GC of 45–55%. The primer details are listed in Table 1. Primer specificity was evaluated by 2.0% agarose gel electrophoresis and as well as with the melt curve analysis.

**Table 1:** Comprehensive details of the finger millet candidate reference genes, primer sequences, amplicon size, melting temperature (Mt), amplification efficiency (E); regression coefficient (R^2^), coefficient of variation (CV). Ah—*Arachis hypogaea*; Ca—*Cicer arietinum*; Ec—*Eleusine coracana*; Pg- *Pennisetum glaucum*, Sb- *Sorghum bicolor*.

S. No	Gene	Name	Source/Acc No.	Primer sequence F / R (5'-3')	Amplicon Size (bp)	Mt	E	R^2^	Average Cq	CV (%)
1	*ACT*	Actin	Ec/HE800188	ATGAGGCCCAGTCCAAGAGA	168	84	1.03	0.997	24.44	8.17
				GGTTCAAAGGGGCTTCAGTG						
2	*CYP*	Cyclophilin	Pg/KM105955	TACAAGGGGTCGAGCTTCCAC	104	89.7	1.08	0.922	27.02	5.0
				TTCTCGCCGTAGATGGACTCC						
3	*EIF4α*	Eukaryotic Initiation factor 4A	Ca/XM_004513380	AGTCACTTCGGCCAGATTACAT	137	84.6	1.01	0.994	21.21	8.42
				AGCAGAGAAAACTCCCACTTGA						
4	*EF1α*	Elongation factor 1-alpha	Ec/HQ202576	GCATGCTCTCCTTGCTTTCA	102	82.7	1.02	0.968	19.91	7.36
				TACTTGGGTGTGGTGGCATC						
5	*GAPDH*	Glyceraldehyde-3-phosphate dehydrogenase	Pg/GQ398107	TGCCTTGCTCCCCTTGCTAA	139	84.6	0.96	0.991	19.57	9.95
				CAGCCCTTCCACCTCTCCAG						
6	*G6PD*	Glucose-6-phosphate 1-dehydrogenase	Ac/EG030635	ACCATTCCAGAGGCTTATGAGC	151	82.5	0.93	0.999	27.93	2.88
				AAGGGAGTGACTTGAACTCTCC						
7	*MACP*	MalonylCoA-Acyl Carrier protein	Sb/XM_002465363	GCATTGAGAACATCGGGGCTT	139	84.6	1.00	0.996	26.51	6.95
				ATGAGTGGAAACTTCGTTCCA						
8	*MDH*	Malate dehydrogenase	Sb/XM_002467034	TGCAGTGGTGGTGAATGGAA	103	83.7	1.01	0.994	26.61	8.04
				GCGTCTTCTCTTCCGACAGC						
9	*S24*	40S ribosomal protein	Pg/KM105960	CCCCAGGAAGTGCTCTGCTA	158	86.1	0.97	0.987	25.79	7.36
				CATCAGCGTCACCCTGAGCA						
10	*PP2A*	Serine/threonine-Protein Phosphatase	Ec/KT824869	GATCGCGTCCAAGAAGTTCC	109	83.1	0.98	0.996	24.32	7.97
				AAGTGTAGCCAGCACCACGA						
11	*PT*	Phosphate transporter protein	Ec/KJ842585	GGCCTCTTCTCCCAGGAGTT	128	87.8	0.95	0.928	29.11	5.56
				TTGATGGCCGTGAAGATGTC						
12	*S21*	Ribosomal protein	Ec-KC894816	ACTTCTACCCCGAGCACACG	152	84.4	0.97	0.994	15.27	13.96
				CGCTTATGACCTCCCCCTCT						
13	*TFIID*	Transcription initiation factor	Ec-KT824872	ACCATGGATGGGTTCTCCAC	163	86.1	0.98	0.959	21.1	7.13
				GATCCTCCTTCCATGCTTGC						
14	*TIP41*	Tonoplast intrinsic proteins -like protein	Ca/XM_004496854	GTTGTACTTCGGGAGAGTTGCT	115	83	0.95	0.956	30.33	2.47
				GGAGCTTCTGGCTTATGATGCT						
15	*β-TUB*	Beta Tubulin	Pg/KM105955	CACCTCCATCCAGGAGATGTT	167	87.4	0.87	0.999	23.21	3.3
				GGTGAACTCCATCTCGTCCA						
16	*UBC*	Ubiquitin Protein Isoform C	Pg/CD724586	TTCAAACCTCCGAAGGTGTCTT	100	81.7	0.88	0.998	23.26	6.07
				GGCTCCACTGCTCTTTAAGAATG						

### Calculation of PCR efficiency

Ten-fold serial dilution of cDNA was used as template for calculating the amplification efficiency (E) of the primer pairs in RT-qPCR with minimum five dilution points. The amplification efficiency (E) and correlation coefficients (R^2^) for each primer set were estimated according to the equation: E = 10^−1/slope^.

### RNA isolation and RT-qPCR

Total RNA of the finger millet samples was isolated from 100 mg of tissue by RNeasy Plant Mini kit (Qiagen, Germany) according to manufacturer’s guidelines. Quantity and quality of RNA were determined by NanoVue plus spectrophotometer (GE health care, USA) and BioAnalyzer (Agilent). Total RNA samples with an absorbance ratio OD 260/280 ranged from 1.9–2.2 were used directly for RT-qPCR analysis. Integrity of RNA was confirmed by running the samples on 1.4% denatured agarose gel electrophoresis. Further, total RNA of all the finger millet experimental samples was diluted to 100 ƞg/μl and it used for RT-qPCR assays. All the RT-qPCR assays were accomplished using SYBR green based quantification assay in a Realplex real-time PCR machine (Eppendrof). A reaction mixture was constituted of 1 μl-RNA (100 ηg), 5-μl one step SYBR RT-PCR buffer 4 (Takara, Japan), 0.4 μl of the prime script one step Enzyme Mix 2 (Takara, Japan) and 400nm of each primer and total volume made to 10 μl with RNase-free H_2_O. The one step RT-qPCR including reverse transcription cycling were as follows: 42 °C for 5 min and 95 °C for 10 s, followed by 40 cycles of denaturing at 95 °C for 15 s, annealing at 62 °C for 15 s with fluorescent signal recording. The dissociation (melt) curve analysis was included after 40 cycles of amplification cycles are completed by heating from 58 °C to 95 °C with fluorescence measured within 20 min. All the RT-qPCR assays were repeated at least three times.

### Samples size and grouping

Experimental samples used in the current study were classified into four sample sets based on their sample nature. The ‘tissue set’ included of five different tissues of the plant development, i.e., seedling, root, leaf, panicle and mature seed of finger millet variety GPU 28 is grown under greenhouse conditions. The ‘abiotic stress set’ comprised of five samples of finger millet grown under different abiotic stresses (Heat, cold, salt, drought and ABA). The ‘genotypes set’ comprised a leaf sample of drought stress and controls of five finger millet genotypes, including three drought tolerant (IE 4073, IE 4797 and GPU 28) and two drought sensitive (IE 5106 and IE 2572). Total 60 samples considering three biological replicates of each sample, from above three sample sets were considered jointly as ‘all sample set’.

### Statistical programs for normalization

Statistical tools named geNorm, NormFinder, BestKeeper and ΔCt were adopted for identifying the best stable candidate reference genes in finger millet. The Cq values of each gene were converted into relative quantities after adjusting them according to their respective PCR efficiencies. The mean values of the relative quantities of the replicates were acquired as the input data for the geNorm and NormFinder tools and data was analysed using genEX Professional software (MultiD Analyses AB). The geNorm tool calculates the expression stability (M) and ranks the genes stability in an order as the lower the M value indicates the higher the stability [19]. The geNorm tool of qBase plus software (ver: 2.4; Biogazelle, Belgium) also relates the pairwise variation (V) of the most stable genes with the rest of the candidate reference genes for efficient normalization in each sample set. A threshold value of 0.15 and less than that indicates no additional reference gene required for normalization in a particular sample set. The NormFinder statistical tool calculates intra- and inter-group variations in gene expression stability and provides ranks accordingly [47]. Genes with the lowermost rank values were considered to be most stably expressed reference gene(s). BestKeeper is an excel-based tool that calculates a Pearson's correlation coefficient for each reference gene, values of p closer to 1.0 indicating greater stability [48]. In the ΔCt tool, the rank order is determined based on pair-wise comparisons of gene-sets and lowest standard deviation indicates highest expression stability of the reference gene [49]. RefFinder, a web-based tool (http://150.216.56.64/referencegene.php) combines all four major statistical tools (geNorm, NormFinder, BestKeeper and comparative ΔCt method) for calculation of the comprehensive ranks.

### Reference gene validation

Abiotic stress inducible *EcNAC1* gene [24] was selected for RT-qPCR data normalization in different genotypes under progressive drought stress. Four finger millet genotypes contrasting with drought stress tolerance viz. susceptible (IE 5106 and IE 2572) and tolerant (IE 4073 and IE 4797) were selected for quantification of the *EcNAC1* gene. Treatments and sample collection were done as mentioned in the plant material and abiotic stress treatments section. Expression of *EcNAC1* gene was normalized with two best (*PT* and *TFIID*) and two least stable finger millet reference genes (*UBC* and *MDH*) selected from the “genotypes set”. The relative expression of *EcNAC1* gene in progressive drought stressed leaf samples was assessed by comparing with respective control samples of same genotype and as well as with the selected combinations of the reference genes using the REST software [52].

## Results

### Selection and cloning of candidate genes

**Fig 1:**
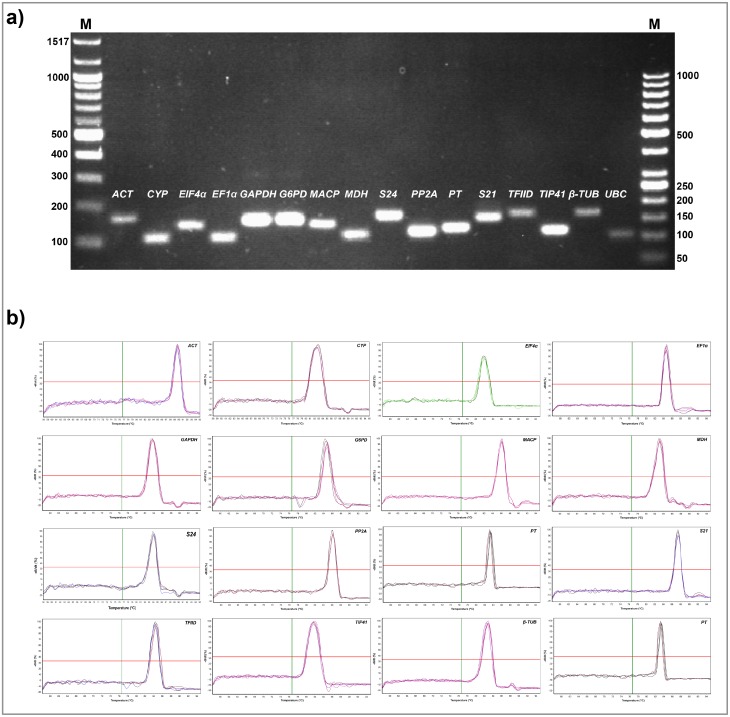
Specificity of finger millet reference gene primer pairs for RT-qPCR amplification. a). The agarose gel (2.0%) electrophoresis displaying a single PCR product with expected sizes for sixteen-finger millet reference genes. M represents the DNA size marker (left side 100 bp DNA marker and right side 50 bp DNA ladder). b). Melt curves of sixteen finger millet reference genes displaying a single and sharp peaks generated from the amplicons.

Six candidate reference genes, including *ACT*, *ELF1α*, *PP2A*, *PT*, *S21* and *TFIID* extracted from the finger millet genome sequence available in the NCBI database were used for the primer designing and further in RT-qPCR study. Remaining ten genes (*β-TUB*, *S24*, *CYP*, *GAPDH*, *EIF4α*, *TIP41*, *UBC*, *G6PD*, *MACP* and *MDH*) were cloned from finger millet cDNA by respective gene specific primers from the various plant sources (Table 1). Amplicons were further verified using agarose gel electrophoresis (Fig 1a) and confirmed by sequencing before being used for RT-qPCR primer designing.

### Primer specificity and PCR efficiency analysis

The amplification specificity of the sixteen finger millet candidate reference genes was studied using regular PCR. The PCR amplification results revealed that all sixteen genes showed distinct and individual amplification of predictable product sizes when resolved on agarose gel (Fig 1a) and melt curve analysis by RT-qPCR (Fig 1b). Predictable product size, distinct PCR amplified fragment, single and sharp melt curve peak and sequencing data representing that the primers had high specificity and were appropriate for RT-qPCR assays. Linear regression coefficient (R^2^) values ranged between 0.922 (*CYP*)—0.999 (*G6PD* and *β-TUB*) and the PCR amplification efficiency in different samples varied from 0.87 (*β-TUB*)—1.08 (*CYP*) (Table 1). Linear regression coefficient (R^2^) and PCR amplification efficiency (E) values were within the acceptable range demonstrating that the primers of the sixteen finger millet reference genes are very specific and applicable for the further analysis.

### Expression analysis of the finger millet reference genes

**Fig 2:**
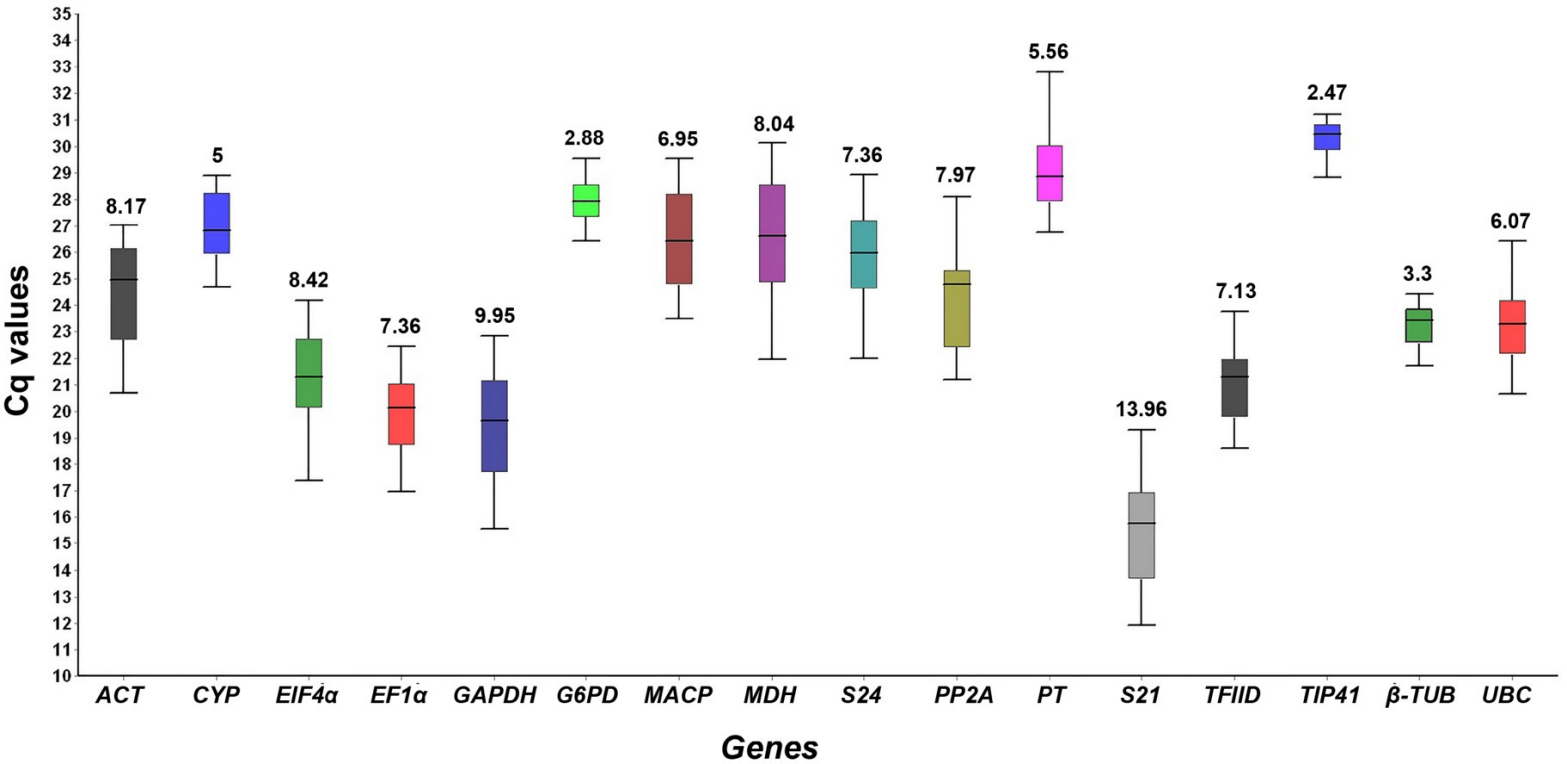
Expression analysis of sixteen finger millet reference genes under different experimental conditions. Box plot representing the Cq distribution of each finger millet reference gene in all the experimental samples. Whisker box denote the highest and lowest Cq values of the each finger millet reference gene in all the experimental samples, and the line across the Whisker box indicates the median value. The coefficient of variation (CV) for each finger millet gene is displayed as a percentage on top of the respective whisker box. The X-axis represents the finger millet genes and the Y-axis represents the Cq value distribution.

The expression analysis of sixteen finger millet reference genes was studied in 21 different experimental samples collected from tissues, abiotic stresses and different genotypes of finger millet. The variable Cq values of sixteen candidate reference genes throughout the experimental samples suggested that their expression levels are highly diverse by the treatments and conditions (Fig 2). The expression of *S21* (CV = 13.96) followed by *GAPDH* (CV = 9.95) were highly affected from sample to sample. In a relative evaluation, a slight range of variable Cq values was detected in case of *TIP41* (CV = 2.47) and *G6PD* (CV = 2.88) empirically suggesting their stable expression under different experimental samples. Regardless of the condition, *S21* was found to be abundant with the lowermost mean Cq value 15.57, and while *TIP41* had highest mean of Cq 30.33 among the tested reference genes throughout the experiments (Fig 2). These implied that the expression of the finger millet reference genes are inconsistent and without any specific pattern across all the experimental conditions. Therefore, there is a need to select a best stable reference gene (s) to normalize the gene expression in a set of samples grouped based on their experimental nature in finger millet.

### Expression stability analysis of the finger millet reference genes

Expression stability of the finger millet reference genes was studied using four different statistical algorithms having distinct principles for stability rankings in order to select the best stable reference genes. In the current study, we detected almost similar tendencies for each condition with subtle variations, which may be attributed by variances in the tools. Because each tool has their respective advantages we adopted four statistical packages in choice of the most and the least stable candidate reference genes. Further precision in the interpretation of the stability ranking made by each statistical tool has been presented individually.

#### geNorm

**Fig 3:**
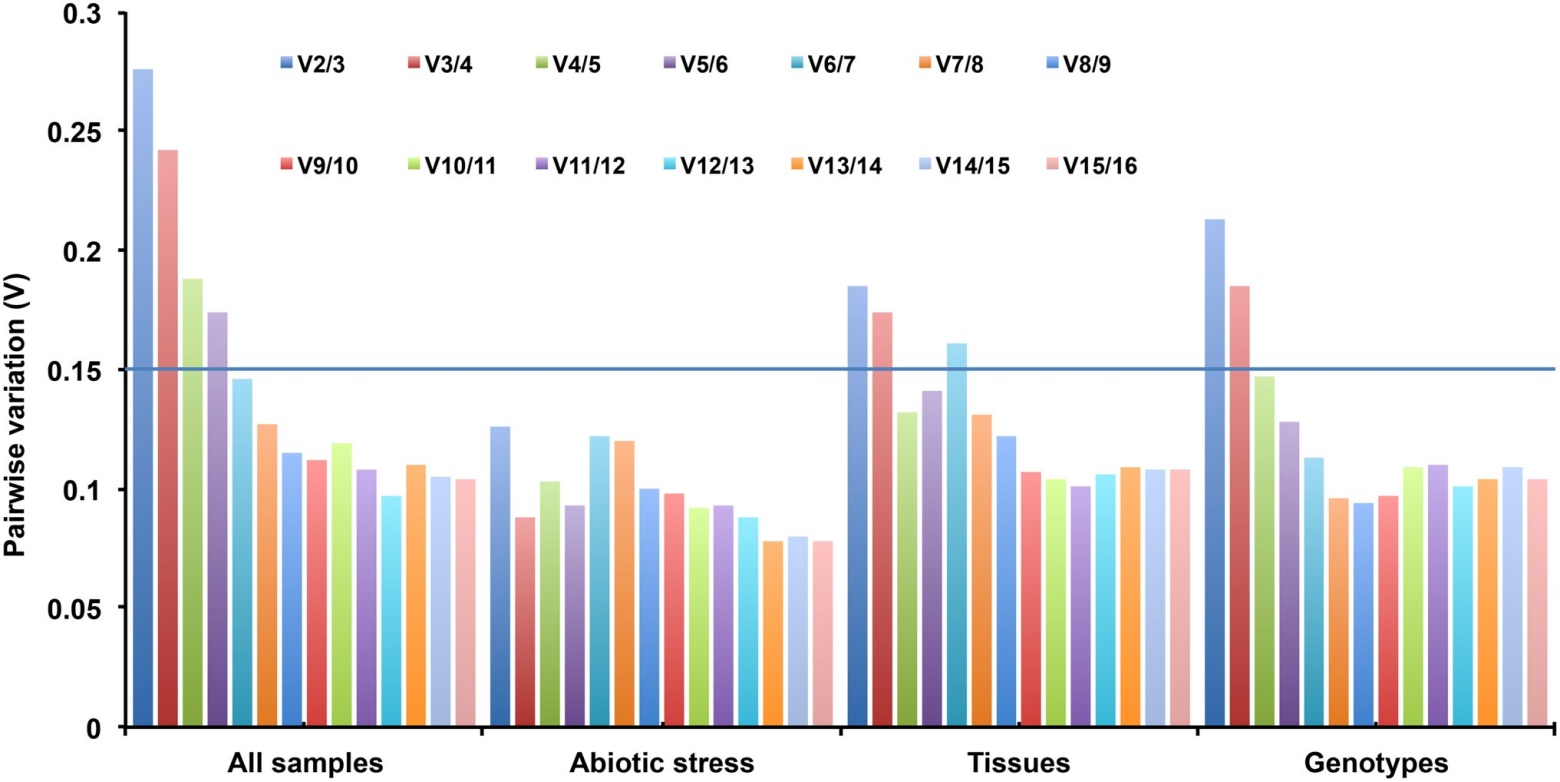
The geNorm analysis for finding the optimal number of finger millet reference genes essential for precise and accurate normalization studies in all four sample sets. The pairwise variation (Vn/Vn+1) was analyzed by the geNorm statistical tool to decide the minimum number of reference genes for precise and accurate RT-qPCR data normalization in each experimental set of finger millet samples. The cutoff value was 0.15; below this value indicates no additional reference gene required for RT-qPCR data normalization.

In geNorm, stability of the sixteen candidate reference genes notably changed in ranking order from sample to sample (Table 2 and S1 Table). In ‘all sample set’ geNorm ranking revealed *MACP* and *PT* (0.85) to be most stable followed by *CYP* (0.96), where *TIP41* (1.47), *S21* (1.42) and *G6PD* (1.37) were the least stable reference genes (S1 Table). Under ‘abiotic stress set’, *CYP*, *S21* (0.25) showed higher stability and *PT* (1.14) and *ACT* (1.10) were the least stable ones. In the ‘tissues set’, *CYP* and *MACP* (0.33) were the most stable and *S21* (1.46) and *G6PD* (1.39) were the least stable genes, whereas in ‘genotypes set’ *MACP* and *S21* (0.45) were the highest in terms of their stability and *MDH* (1.25) and *UBC* (1.17) as the least stable amongst all. The pairwise variation V2/3 value was greater than 0.15 in ‘all-samples set’ and less than 0.15 in the case of ‘abiotic stress set’ (V2/3 = 0.13) (Fig 3). These results implied that use of more than two best stable finger millet reference genes together would be required for normalization studies in genotypes (V4/5 = 0.15), tissues (V4/5 = 0.13) and all sample sets (V6/7 = 0.15) (Fig 3).

**Table 2:** Expression stability rankings order of all sixteen- finger millet candidate references genes validated from five different tools: Delta Ct (ΔCT), geNorm (GN), NormFinder (NF), BestKeeper (BK), and RefFinder (RF).

	All Samples					Abiotic stress					Tissues					Genotypes				
Rank	ΔCT	GN	NF	BK	RF	ΔCT	GN	NF	BK	RF	ΔCT	GN	NF	BK	RF	ΔCT	GN	NF	BK	RF
**1**	*CYP*	*MACP*	*CYP*	*TIP41*	*CYP*	*β-TUB*	*CYP*	*β-TUB*	*β-TUB*	*β-TUB*	*MACP*	*CYP*	*MACP*	*G6PD*	*MACP*	*PT*	*MACP*	*PT*	*TIP41*	*PT*
**2**	*EF1α*	*PT*	*EF1α*	*G6PD*	*β-TUB*	*CYP*	*S21*	*CYP*	*G6PD*	*CYP*	*CYP*	*MACP*	*CYP*	*β-TUB*	*CYP*	*TFIID*	*S21*	*EF1α*	*G6PD*	*TFIID*
**3**	*β- TUB*	*CYP*	*β-TUB*	*β-TUB*	*EF1α*	*S21*	*β-TUB*	*S21*	*CYP*	*S21*	*EF1α*	*EF1α*	*EF1α*	*TIP41*	*EF1α*	*EF1α*	*TFIID*	*TFIID*	*β-TUB*	*MACP*
**4**	*PT*	*β-Tub*	*PT*	*UBC*	*PT*	*G6PD*	*G6PD*	*G6PD*	*UBC*	*G6PD*	*TIP41*	*MDH*	*TIP41*	*EIF4a*	*TIP41*	*CYP*	*PT*	*CYP*	*CYP*	*EF1α*
**5**	*MACP*	*TFIID*	*TFIID*	*EF1a*	*MACP*	*EF1α*	*UBC*	*EF1α*	*S21*	*UBC*	*β-TUB*	*UBC*	*β-TUB*	*PP2A*	*β-TUB*	*MACP*	*PP2A*	*GAPDH*	*PT*	*S21*
**6**	*EIF4α*	*EF1α*	*EIF4α*	*CYP*	*TFIID*	*UBC*	*EF1α*	*UBC*	*EF1a*	*EF1α*	*GAPDH*	*EIF4α*	*EIF4α*	*UBC*	*EIF4α*	*S21*	*ACT*	*MACP*	*TFIID*	*CYP*
**7**	*TFIID*	*EIF4α*	*MACP*	*TFIID*	*EIF4α*	*PP2A*	*MDH*	*PP2A*	*ACT*	*MDH*	*MDH*	*GAPDH*	*GAPDH*	*MACP*	*G6PD*	*PP2A*	*GAPDH*	*S21*	*MACP*	*TIP41*
**8**	*GAPDH*	*GAPDH*	*GAPDH*	*PT*	*TIP41*	*MDH*	*PP2A*	*MDH*	*TIP41*	*PP2A*	*EIF4α*	*ACT*	*PP2A*	*CYP*	*UBC*	*GAPDH*	*EF1α*	*PP2A*	*EF1a*	*β-TUB*
**9**	*PP2A*	*PP2A*	*PP2A*	*EIF4a*	*G6PD*	*TIP41*	*EIF4α*	*TIP41*	*MDH*	*TIP41*	*ACT*	*TIP41*	*ACT*	*PT*	*GAPDH*	*β-TUB*	*CYP*	*ACT*	*UBC*	*PP2A*
**10**	*ACT*	*ACT*	*ACT*	*S24*	*GAPDH*	*EIF4α*	*S24*	*EIF4α*	*TFIID*	*EIF4α*	*PP2A*	*β-TUB*	*MDH*	*S21*	*MDH*	*ACT*	*β-TUB*	*β-TUB*	*S21*	*GAPDH*
**11**	*S24*	*S24*	*S24*	*MACP*	*UBC*	*TFIID*	*TIP41*	*TFIID*	*PP2A*	*TFIID*	*UBC*	*PT*	*UBC*	*EF1a*	*PP2A*	*S24*	*EIF4α*	*S24*	*PP2A*	*G6PD*
**12**	*UBC*	*MDH*	*UBC*	*PP2A*	*PP2A*	*MACP*	*GAPDH*	*MACP*	*EIF4a*	*ACT*	*PT*	*PP2A*	*PT*	*ACT*	*ACT*	*EIF4α*	*S24*	*EIF4α*	*S24*	*ACT*
**13**	*MDH*	*UBC*	*G6PD*	*GAPDH*	*S24*	*GAPDH*	*TFIID*	*GAPDH*	*GAPDH*	*GAPDH*	*TFIID*	*TFIID*	*TFIID*	*S24*	*PT*	*TIP41*	*TIP41*	*UBC*	*GAPDH*	*S24*
**14**	*G6PD*	*G6PD*	*MDH*	*ACT*	*ACT*	*S24*	*MACP*	*S24*	*MACP*	*MACP*	*S24*	*S24*	*S24*	*GAPDH*	*TFIID*	*UBC*	*G6PD*	*TIP41*	*EIF4a*	*EIF4α*
**15**	*S21*	*S21*	*S21*	*S21*	*MDH*	*ACT*	*ACT*	*ACT*	*S24*	*S24*	*G6PD*	*G6PD*	*G6PD*	*MDH*	*S24*	*G6PD*	*UBC*	*G6PD*	*ACT*	*UBC*
**16**	*TIP41*	*TIP41*	*TIP41*	*MDH*	*S21*	*PT*	*PT*	*PT*	*PT*	*PT*	*S21*	*S21*	*S21*	*TFIID*	*S21*	*MDH*	*MDH*	*MDH*	*MDH*	*MDH*

#### NormFinder

NormFinder algorithm ranked *CYP* (0.53) and *EF1a* (0.69) as the top two most stable genes having steady expression across experiments, while the expression of *TIP41* (1.55) and *S21* (1.53) were highly altered and least in the stability (Table 2 and S2 Table). Under abiotic stress, *PT* (1.23) and *ACT* (1.21) were least stable, whereas *β-TUB* (0.26) and *CYP* (*0*.*26*) were the most stable ones ranking higher than *S21 (0*.*494)*. *MACP* (0.24) and *CYP* (0.35) were ranked higher in ‘tissue set’, whereas *S21* (1.68) and *G6PD* (1.65) were least stable as per their rankings. Gene expression studies in ‘genotypes set’ showed *PT* (0.31) and *EF1α* (0.53) in the top 2 slots whereas *MDH* (1.61) and *G6PD* (1.34) were the least stable finger millet reference genes in this study (S2 Table).

#### BestKeeper

BestKeeper determines rankings based on the standard deviation (SD) values, which is inversely proportional to the expression stability of the genes. *TIP41* (SD = 0.6) followed by *β-TUB* and *G6PD* (SD = 0.65) were most stably expressed and ranked high across all experimental samples, in contrast to, *MDH* expression that revealed significant variation (SD = 1.72) (Table 2 and S3 Table). Intriguingly, gene-expression stability of *β-TUB* (SD = 0.07) was least affected in ‘abiotic stress set’, whereas *S24* and *PT* (SD = 0.99) were the least stable genes. In ‘tissue set’, *G6PD* and *β-TUB* (SD = 0.47) displayed most stable expression, while *TFIID* (SD = 1.83) and *MDH* (SD = 1.77) were found to be the least stable ones. In the ‘genotype’ set, *TIP41* (SD = 0.31) and *G6PD* (SD = 0.42) were placed as the best reference genes followed by *β-TUB* (SD = 0.53), while *MDH* (SD = 1.74) and *ACT* (SD = 1.58) as least useful (S3 Table).

#### Delta CT (ΔCt)

The ΔCt algorithm recognized *CYP* (1.19) and *EF1α* (1.25) as the best stable finger millet reference genes in ‘all samples set’ (Table 2 and S4 Table) whereas *TIP41* (1.83) and *S21* (1.81) were the least stable genes. In ‘tissues set’, *S21* (1.93) and *G6PD* (1.89) was the least stable, whereas *MACP* (1.08) and *CYP* (1.1) were most stable reference genes. The *β-TUB* (0.85) and *CYP* (0.86) were ranked most stable in ‘abiotic stress set’, while *PT* (1.44) and *ACT* (1.41) were the least stable genes. ΔCt method in ‘genotypes set’ identified *PT* (0.97) and *TFIID* (1.04) as most stable while *MDH* (1.76) and *G6PD* (1.56) were least stable of the lot (S4 Table).

#### RefFinder

**Fig 4:**
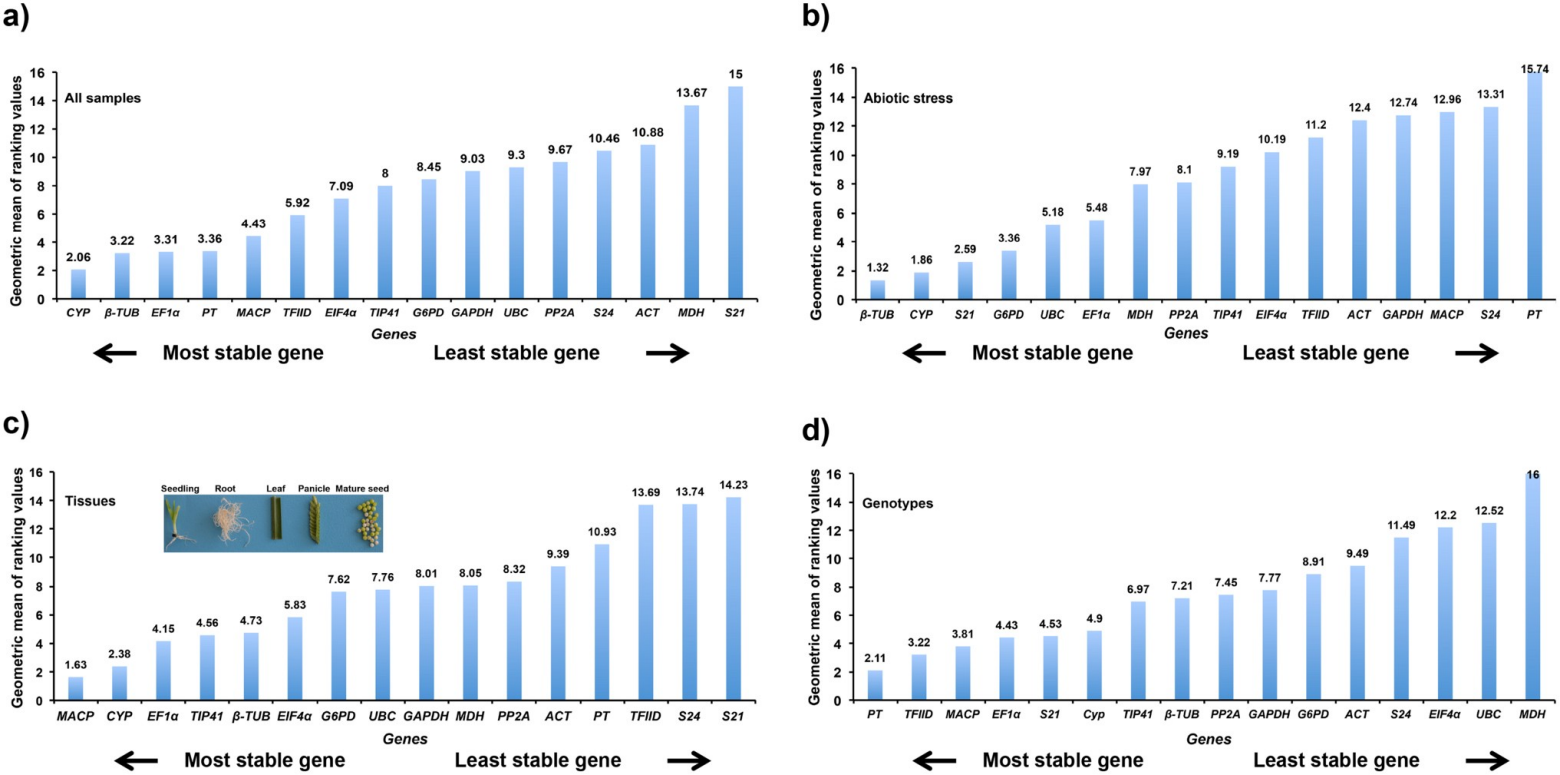
Expression stability ranking of the sixteen finger millet reference genes in various sample sets (a-d) using RefFinder analysis. The X-axis represents the genes and the geometric mean values were specified on Y-axis; a lesser value indicates more stability and the higher value indicates the least stability of evaluated finger millet reference genes.

RefFinder analysis revealed that, the expression of *CYP* (2.06) and *β-TUB* (3.22) were least affected by variations in ‘all sample set’, whereas *MDH* (13.67) and *S21* (15) displayed highest variations (Fig 4a, Table 2 and S5 Table). *PT* (15.74) and *S24* (13.31) were extremely unstable under abiotic stress, whereas *β-TUB* (1.32) and *CYP* (1.86) showed much higher stability among the sixteen candidate reference genes (Fig 4b and Table 2). Similarly, the expression of *MACP* (1.63) and *CYP* (2.38) varied least among the ‘tissue set’, while *S21* (14.23) and *S24* (13.74) varied drastically (Fig 4c and S5 Table). A narrow range of variability in stability of *PT* (2.11) and *TFIID* (3.22) across the ‘genotypes set’ suggested that they’re relatively more stable expression, while *MDH* (16) and *UBC* (12.52) shown highest variability in expression (Fig 4d and S5 Table).

### Validation of the reference genes

**Fig 5:**
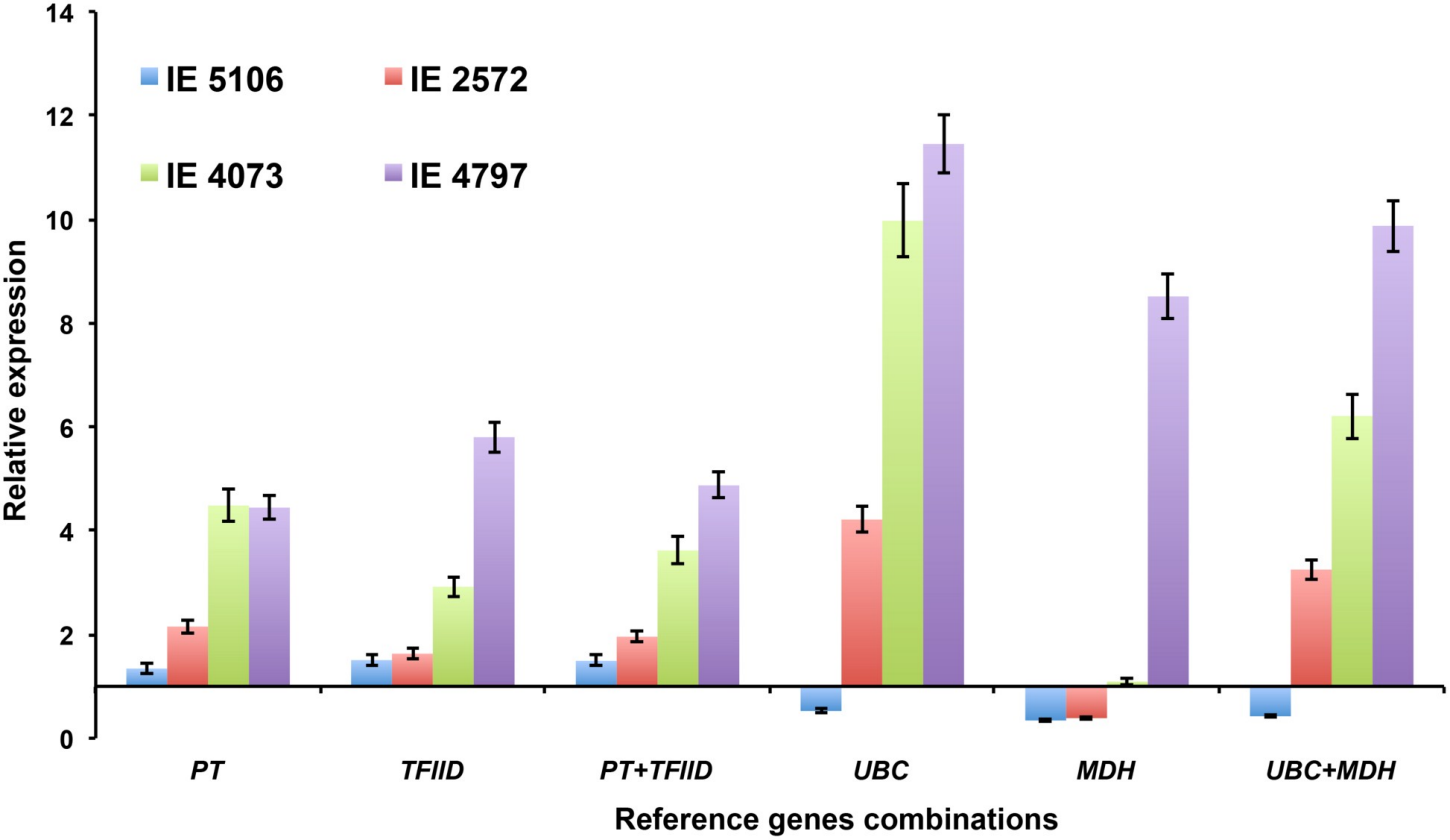
Validation of the best and least stable finger millet reference genes. The relative expression level of *EcNAC1* gene in leaf tissues of drought susceptible (IE 5106 and IE 2572) and tolerant (IE 4073 and IE 4797) genotypes under drought stress, using most and least stable reference genes nominated from the RefFinder tool. *EcNAC1* gene transcript levels were normalized in the individual and combined manner with both most and least stable reference genes. Value represents the mean of three technical and biological replicates. Standard error bars are shown.

To analyze the transcript level of *EcNAC1* gene under drought stress in different genotypes, we selected two best stable (*PT* and *TFIID*) and least stable (*UBC* and *MDH*) finger millet reference genes identified in the present study from the ‘genotype set’ and were used for validation of the normalized results. *PT* and *TFIID* individually or combined showed higher stability in RT-qPCR data compared to least stable *UBC* and *MDH* both independently and in a combination (Fig 5). Normalization with *PT* and *TFIID* genes produced more consistent and comparable results in drought susceptible (IE 5106 and IE 2572) and tolerant (IE 4073, IE 4797) genotypes which clearly showed differential expression of transcripts of *EcNAC1* gene (Fig 5). *EcNAC1* gene expression was higher in drought tolerant genotypes (IE 4073 and IE 4797) when compared with susceptible ones (IE 5106 and IE 2572) and followed a similar trend when normalized with best stable reference genes. In contrast, normalizations using the lesser stable reference genes showed significant variation and inconsistency in the results with discrepancies in the drought tolerant and susceptible genotypes (Fig 5).

## Discussion

The recent draft genome sequence of *Eleusine coracana* (L.) provides an excellent genomic resource for the research community that should facilitate a holistic understanding of the genetic basis of its innate nutritional potentials and drought stress tolerance [8]. A total of 85,243 genes from this genome sequencing data, predicted as stress related transcription factors/genes, calcium transporters accumulation genes and C_4_ photosynthetic pathway genes, make it worthwhile to validate the function of the genes for understanding their role in various biological processes. So far most of the RT-qPCR analyses in finger millet have used reference genes either from heterologous plant species or from the native finger millet without any systematic normalization validation studies [23–34]. This becomes a serious hindrance in precise analysis of the RT-qPCR data and requires a comprehensive evaluation of the reference genes in finger millet under various experimental conditions.

In the this study, sixteen candidate reference genes (Table 1) were used to analyze their expression-stability under various experimental conditions referred to as ‘genotypes’, ‘tissues’, ‘abiotic stress’ and ‘all samples’ sets. The gene-expression data clearly indicate the effect of treatments on the stability of reference genes. The expression stability of all finger millet genes was not affected by each treatment, few genes got affected and others have not irrespective of the conditions (Fig 2). Together, the results reported earlier imply that no single reference gene is stable across all the conditions and treatments [20,21]. The best stable reference gene (s) for each condition were indicated by its order of rank using different statistical algorithms (Table 2 and S1–S5 Tables). Although expression stability ranking order of the finger millet reference genes was not same in each method, a realistic consent undoubtedly implied the top order finger millet reference genes for each condition (Fig 4). For example, *CYP* and *EF1α* were positioned as best stable reference genes by NormFinder and ΔCt method for studying gene expression in all samples set, while geNorm ranked *MACP* as the best and BestKeeper placed *TIP41* as most stable gene. The subtle variation in ranking order of the top listed reference genes could be endorsed to difference in tools of the software and sensitivities towards the co-expressed reference genes [43]. It was also evident that the top-ranked stable reference gene for one condition may not appropriate to the other condition. Hence, it was vital to find the stable reference genes that show adequate for RT-qPCR assay, if not the uppermost stability in expression within and across the conditions can be considered.

RefFinder study suggested *CYP*, *β-TUB*, and *EF1α* as the top stable finger millet reference genes for their stable expression in most of the conditions. These interpretations are in correlation with earlier findings where *CYP* and *EF1α* were described as most steadily expressed reference genes under diverse experimental conditions of Petunia and *Vicia faba* [31,53,54]. In alternative study, *CYP* gene was revealed to express stably in different developmental stages and *EF1α* was revealed to be least variant in expression in the samples collected at different time intervals in soybean [55]. Additionally, *CYP* gene was recommended as best stable gene in combination with other three reference genes for normalization studies in soybean [56]. The *EF1α* gene was also reported as best stable reference gene under various experimental conditions in different plant species including chinese cabbage [57], pearl millet [20], and potato [58]. The third most stable reference gene *β-TUB* was also recommended as best stable in many plant species. The genes *β-TUB* and *EF1α* were suggested in combinations with *ACT* gene for gene expression analysis under biotic and abiotic stress treated samples of *Vigna mungo* [59], and Musa [60]. The *β-TUB* gene was also reported as the most stable under leaf senescence conditions in sunflower [61]. There has been an extensive discussion about the optimal number of most stable reference genes required for RT-qPCR data analysis. For preventing errors and increasing the accuracy in normalization process, investigators have proved use of more than one reference genes instead of single gene [20,21,41,42]. In our present study, geNorm pair-wise analysis implies use of more than two stably reference genes in achieving accuracy during RT-qPCR data normalization for all the experimental samples except abiotic stress, where two reference genes would be beneficial (Fig 3). Therefore, we propose the use of two and more reference genes in combinations for normalizing of gene expression assays under different experimental samples in finger millet.

In the present study *EcNAC1* a drought and salinity stress responsive gene [24], was selected as experimental gene for the validation of normalization results (Fig 5). *EcNAC1* gene was expressed high in tolerant genotypes (IE 4073 and IE 4797) in comparison with susceptible genotypes (IE 5106 and IE 2572) and followed distinctive pattern in expression when normalized with the stable reference gene (s) (*PT* and *TFIID*). In contrary, conflict and inconsistency was observed in expression levels when uses least stable reference gene (s) (*UBC* and *MDH*) (Fig 5). In summary, we conclude that the reference genes validated in the present study were appropriate for data normalization in RT-qPCR studies under various experimental conditions in finger millet.

## Conclusion

Owing to the agronomic significance of *Eleusine coracana* as future food security crop, gene expression studies would endure to represent a significant part of basic and functional genomics research in finger millet. Therefore, establishing standardized reference genes for RT-qPCR studies in *E*. *coracana* would assist the peer researchers working in finger millet functional genomics. The present study reveals that the traditional and heterologous plant source candidate reference genes may not be appropriate for their direct use in RT-qPCR data normalization studies without systematic investigational validation across the experimental conditions. The stability rank order of the finger millet reference genes from all the experimental conditions implied that no single reference gene could be used perfectly for all the experimental conditions. In summary, we recommend the use of *CYP*, *β-TUB* and *EF1α*, preferably in combination for robust normalization of RT-qPCR data under most of the experimental conditions. The present study is helpful for undertaking the future RT-qPCR based expression studies in the finger millet.

## Supporting information

S1 Table geNorm stability ranks based on gene expression stability (M) values, where lower value indicates more stable reference gene. (DOCX)

S2 Table NormFinder stability ranks based on gene expression stability (M) values, where lower value indicates more stable reference gene. (DOCX)

S3 Table Best Keeper stability ranks based on standard deviation (SD) which is inversely proportional to the stability of the expression. (DOCX)

S4 Table DeltaCT stability ranks based on average standard deviation (SD) which is inversely proportional to the stability of the expression. (DOCX)

S5 Table RefFinder comprehensive ranks based on geometric mean values calculated from the four algorithms (geNorm, NormFinder, BestKeeper and ΔCt); geometric mean value is inversely proportional to the stability of the expression. (DOCX)

## Abbreviations

CYP: Cyclophilin/Peptidylprolyl Isomerase
EIF4α: Eukaryotic Initiation factor 4A
MACP: Malonyl CoA-Acyl Carrier protein
PT: Phosphate transporter protein
RT-qPCR: Reverse Transcription Quantitative PCR
TFIID: Transcription initiation factor
