# Supplementary material for: Comprehensive evaluation of candidate reference genes for real-time quantitative PCR (RT-qPCR) data normalization in nutri-cereal finger millet [Eleusine Coracana (L.)]
Source: PLoS One. 2018 Oct 15;13(10):e0205668. doi: 10.1371/journal.pone.0205668 (PMC6188778; doi:10.1371/journal.pone.0205668)
Supplement: S1 Table — (DOCX) [file pone.0205668.s001.docx]

| All samples | | Abiotic stress | | Tissues | | Genotypes | |
| --- | --- | --- | --- | --- | --- | --- | --- |
| Gene | **Stability value (M)** | **Gene** | **Stability value (M)** | **Gene** | **Stability value (M)** | **Gene** | **Stability value (M)** |
| *MACP\|PT* | 0.852 | *CYP\|S21* | 0.246 | *CYP\|MACP* | 0.327 | *MACP\|S21* | 0.455 |
| *CYP* | 0.958 | *β-TUB* | 0.344 | *EF1a* | 0.493 | *TFIID* | 0.594 |
| *β-TUB* | 1.028 | *G6PD* | 0.375 | *MDH* | 0.631 | *PT* | 0.646 |
| *TFIID* | 1.062 | *UBC* | 0.451 | *UBC* | 0.778 | *PP2A* | 0.682 |
| *EF1α* | 1.095 | *EF1a* | 0.505 | *EIF4α* | 0.846 | *ACT* | 0.743 |
| *EIF4α* | 1.142 | *MDH* | 0.617 | *GAPDH* | 0.932 | *GAPDH* | 0.791 |
| *GAPDH* | 1.176 | *PP2A* | 0.721 | *ACT* | 0.994 | *EF1α* | 0.823 |
| *PP2A* | 1.2 | *EIF4α* | 0.789 | *TIP41* | 1.056 | *Cyc* | 0.873 |
| *ACT* | 1.224 | *S24* | 0.849 | *β-TUB* | 1.101 | *β-TUB* | 0.907 |
| *S24* | 1.261 | *TIP41* | 0.905 | *PT* | 1.155 | *EIF4α* | 0.961 |
| *MDH* | 1.291 | *GAPDH* | 0.965 | *PP2a* | 1.2 | *S24* | 0.994 |
| *UBC* | 1.325 | *TFIID* | 1.015 | *TFIID* | 1.248 | *TIP41* | 1.064 |
| *G6PD* | 1.367 | *MACP* | 1.054 | *S24* | 1.313 | *G6PD* | 1.123 |
| *S21* | 1.421 | *ACT* | 1.096 | *G6PD* | 1.394 | *UBC* | 1.175 |
| *TIP41* | 1.472 | *PT* | 1.138 | *S21* | 1.461 | *MDH* | 1.248 |

**S1 Table. geNorm stability ranks based on gene expression stability (M) values, where lower value indicates more stable reference gene.**
