# Supplementary material for: Comprehensive evaluation of candidate reference genes for real-time quantitative PCR (RT-qPCR) data normalization in nutri-cereal finger millet [Eleusine Coracana (L.)]
Source: PLoS One. 2018 Oct 15;13(10):e0205668. doi: 10.1371/journal.pone.0205668 (PMC6188778; doi:10.1371/journal.pone.0205668)
Supplement: S2 Table — (DOCX) [file pone.0205668.s002.docx]

**S2 Table. NormFinder stability ranks based on gene expression stability (M) values, where lower value indicates more stable reference gene.**

| All samples | | Abiotic stress | | Tissues | | Genotypes | |
| --- | --- | --- | --- | --- | --- | --- | --- |
| Gene | **Stability value (M)** | **Gene** | **Stability value (M)** | **Gene** | **Stability value (M)** | **Gene** | **Stability value (M)** |
| *CYP* | 0.528 | *β-TUB* | 0.259 | *MACP* | 0.244 | *PT* | 0.309 |
| *EF1α* | 0.694 | *CYP* | 0.264 | *CYP* | 0.347 | *EF1α* | 0.532 |
| *β-TUB* | 0.779 | *S21* | 0.494 | *EF1α* | 0.552 | *TFIID* | 0.534 |
| *PT* | 0.803 | *G6PD* | 0.595 | *TIP41* | 0.675 | *CYP* | 0.563 |
| *TFIID* | 0.878 | *EF1α* | 0.621 | *β-TUB* | 0.842 | *GAPDH* | 0.752 |
| *EIF4α* | 0.891 | *UBC* | 0.667 | *EIF4α* | 0.982 | *MACP* | 0.752 |
| *MACP* | 0.893 | *PP2A* | 0.745 | *GAPDH* | 0.987 | *S21* | 0.761 |
| *GAPDH* | 0.962 | *MDH* | 0.795 | *PP2A* | 1.024 | *PP2A* | 0.77 |
| *PP2A* | 1.07 | *TIP41* | 0.866 | *ACT* | 1.038 | *ACT* | 0.875 |
| *ACT* | 1.079 | *EIF4α* | 0.91 | *MDH* | 1.061 | *β-TUB* | 0.884 |
| *S24* | 1.156 | *TFIID* | 0.983 | *UBC* | 1.131 | *S24* | 0.903 |
| *UBC* | 1.24 | *MACP* | 1.015 | *PT* | 1.144 | *EIF4α* | 0.919 |
| *G6PD* | 1.248 | *GAPDH* | 1.029 | *TFIID* | 1.321 | *UBC* | 1.242 |
| *MDH* | 1.271 | *S24* | 1.123 | *S24* | 1.546 | *TIP41* | 1.266 |
| *S21* | 1.534 | *ACT* | 1.209 | *G6PD* | 1.655 | *G6PD* | 1.342 |
| *TIP41* | 1.546 | *PT* | 1.233 | *S21* | 1.682 | *MDH* | 1.605 |
