# Supplementary material for: Comprehensive evaluation of candidate reference genes for real-time quantitative PCR (RT-qPCR) data normalization in nutri-cereal finger millet [Eleusine Coracana (L.)]
Source: PLoS One. 2018 Oct 15;13(10):e0205668. doi: 10.1371/journal.pone.0205668 (PMC6188778; doi:10.1371/journal.pone.0205668)
Supplement: S3 Table — (DOCX) [file pone.0205668.s003.docx]

**S3 Table . Best Keeper stability ranks based on standard deviation (SD) which is inversely proportional to the stability of the expression.**

| All samples | | Abiotic stress | | Tissues | | Genotypes | |
| --- | --- | --- | --- | --- | --- | --- | --- |
| Gene | **Std dev** | **Gene** | **Std dev** | ***Gene*** | **Std dev** | **Gene** | **Std dev** |
| *TIP41* | 0.6 | *β-TUB* | 0.07 | *G6PD* | 0.47 | *TIP41* | 0.31 |
| *β-TUB* | 0.65 | *G6PD* | 0.24 | *β-TUB* | 0.47 | *G6PD* | 0.42 |
| *G6PD* | 0.65 | *CYP* | 0.26 | *TIP41* | 0.68 | *β-TUB* | 0.53 |
| *UBC* | 1.15 | *UBC* | 0.31 | *EIF4α* | 0.91 | *CYP* | 0.68 |
| *EF1α* | 1.18 | *S21* | 0.38 | *PP2A* | 1.07 | *PT* | 0.92 |
| *CYP* | 1.2 | *EF1α* | 0.47 | *UBC* | 1.08 | *TFIID* | 1.02 |
| *TFIID* | 1.23 | *ACT* | 0.57 | *MACP* | 1.11 | *MACP* | 1.17 |
| *PT* | 1.26 | *TIP41* | 0.66 | *CYP* | 1.18 | *EF1α* | 1.18 |
| *S24* | 1.4 | *MDH* | 0.71 | *PT* | 1.25 | *UBC* | 1.18 |
| *EIF4α* | 1.4 | *TFIID* | 0.72 | *S21* | 1.25 | *S21* | 1.26 |
| *MACP* | 1.43 | *PP2A* | 0.77 | *EF1α* | 1.32 | *PP2A* | 1.32 |
| *PP2A* | 1.51 | *EIF4α* | 0.83 | *ACT* | 1.37 | *S24* | 1.33 |
| *GAPDH* | 1.54 | *GAPDH* | 0.85 | *S24* | 1.48 | *GAPDH* | 1.42 |
| *ACT* | 1.62 | *MACP* | 0.95 | *GAPDH* | 1.73 | *EIF4α* | 1.47 |
| *S21* | 1.69 | *S24* | 0.99 | *MDH* | 1.77 | *ACT* | 1.58 |
| *MDH* | 1.72 | *PT* | 0.99 | *TFIID* | 1.83 | *MDH* | 1.74 |
