# Supplementary material for: Comprehensive evaluation of candidate reference genes for real-time quantitative PCR (RT-qPCR) data normalization in nutri-cereal finger millet [Eleusine Coracana (L.)]
Source: PLoS One. 2018 Oct 15;13(10):e0205668. doi: 10.1371/journal.pone.0205668 (PMC6188778; doi:10.1371/journal.pone.0205668)
Supplement: S4 Table — (DOCX) [file pone.0205668.s004.docx]

**S4 Table. DeltaCT stability ranks based on average standard deviation (SD) which is inversely proportional to the stability of the expression.**

| All samples | | Abiotic stress | | Tissues | | Genotypes | |
| --- | --- | --- | --- | --- | --- | --- | --- |
| Gene | **Avg of STDEV** | **Gene** | **Avg of STDEV** | **Gene** | **Avg of STDEV** | **Gene** | **Avg of STDEV** |
| *CYP* | 1.19 | *β-TUB* | 0.85 | *MACP* | 1.08 | *PT* | 0.97 |
| *EF1α* | 1.25 | *CYP* | 0.86 | *CYP* | 1.1 | *TFIID* | 1.04 |
| *β-TUB* | 1.31 | *S21* | 0.93 | *EF1α* | 1.17 | *EF1α* | 1.05 |
| *PT* | 1.33 | *G6PD* | 0.97 | *TIP41* | 1.27 | *CYP* | 1.09 |
| *MACP* | 1.37 | *EF1α* | 0.99 | *β-TUB* | 1.34 | *MACP* | 1.12 |
| *EIF4α* | 1.37 | *UBC* | 1.01 | *GAPDH* | 1.39 | *S21* | 1.13 |
| *TFIID* | 1.38 | *PP2A* | 1.1 | *MDH* | 1.42 | *PP2A* | 1.15 |
| *GAPDH* | 1.43 | *MDH* | 1.11 | *EIF4α* | 1.42 | *GAPDH* | 1.16 |
| *PP2A* | 1.47 | *TIP41* | 1.18 | *ACT* | 1.43 | *β-TUB* | 1.21 |
| *ACT* | 1.49 | *EIF4α* | 1.2 | *PP2A* | 1.46 | *ACT* | 1.22 |
| *S24* | 1.53 | *TFIID* | 1.25 | *UBC* | 1.5 | *S24* | 1.24 |
| *UBC* | 1.59 | *MACP* | 1.28 | *PT* | 1.54 | *EIF4a* | 1.26 |
| *MDH* | 1.6 | *GAPDH* | 1.31 | *TFIID* | 1.65 | *TIP41* | 1.5 |
| *G6PD* | 1.6 | *S24* | 1.34 | *S24* | 1.8 | *UBC* | 1.52 |
| *S21* | 1.81 | *ACT* | 1.41 | *G6PD* | 1.89 | *G6PD* | 1.56 |
| *TIP41* | 1.83 | *PT* | 1.44 | *S21* | 1.93 | *MDH* | 1.76 |
