# Supplementary material for: Comprehensive evaluation of candidate reference genes for real-time quantitative PCR (RT-qPCR) data normalization in nutri-cereal finger millet [Eleusine Coracana (L.)]
Source: PLoS One. 2018 Oct 15;13(10):e0205668. doi: 10.1371/journal.pone.0205668 (PMC6188778; doi:10.1371/journal.pone.0205668)
Supplement: S5 Table — (DOCX) [file pone.0205668.s005.docx]

**S Table 5.** **RefFinder comprehensive ranks based on geometric mean values calculated from the four algorithms (geNorm, NormFinder, BestKeeper and ∆Ct); geometric mean value is inversely proportional to the stability of the expression.**

| All samples | | Abiotic stress | | Tissues | | Genotypes | |
| --- | --- | --- | --- | --- | --- | --- | --- |
| Gene | **Geomean of ranking values** | **Gene** | **Geomean of ranking values** | **Gene** | **Geomean of ranking values** | **Gene** | **Geomean of ranking values** |
| *CYP* | 2.06 | *β-TUB* | 1.32 | *MACP* | 1.63 | *PT* | 2.11 |
| *β-TUB* | 3.22 | *CYP* | 1.86 | *CYP* | 2.38 | *TFIID* | 3.22 |
| *EF1α* | 3.31 | *S21* | 2.59 | *EF1α* | 4.15 | *MACP* | 3.81 |
| *PT* | 3.36 | *G6PD* | 3.36 | *TIP41* | 4.56 | *EF1α* | 4.43 |
| *MACP* | 4.43 | *UBC* | 5.18 | *β-TUB* | 4.73 | *S21* | 4.53 |
| *TFIID* | 5.92 | *EF1α* | 5.48 | *EIF4α* | 5.83 | *CYP* | 4.9 |
| *EIF4α* | 7.09 | *MDH* | 7.97 | *G6PD* | 7.62 | *TIP41* | 6.97 |
| *TIP41* | 8 | *PP2A* | 8.1 | *UBC* | 7.76 | *β-TUB* | 7.21 |
| *G6PD* | 8.45 | *TIP41* | 9.19 | *GAPDH* | 8.01 | *PP2A* | 7.45 |
| *GAPDH* | 9.03 | *EIF4α* | 10.19 | *MDH* | 8.05 | *GAPDH* | 7.77 |
| *UBC* | 9.3 | *TFIID* | 11.2 | *PP2A* | 8.32 | *G6PD* | 8.91 |
| *PP2A* | 9.67 | *ACT* | 12.4 | *ACT* | 9.39 | *ACT* | 9.49 |
| *S24* | 10.46 | *GAPDH* | 12.74 | *PT* | 10.93 | *S24* | 11.49 |
| *ACT* | 10.88 | *MACP* | 12.96 | *TFIID* | 13.69 | *EIF4α* | 12.2 |
| *MDH* | 13.67 | *S24* | 13.31 | *S24* | 13.74 | *UBC* | 12.52 |
| *S21* | 15 | *PT* | 15.74 | *S21* | 14.23 | *MDH* | 16 |
